# Editorial: Thyroid nodule evaluation: current, evolving, and emerging tools

**DOI:** 10.3389/fendo.2023.1276323

**Published:** 2023-09-05

**Authors:** Jeffrey R. Garber, Andrea Frasoldati, Vivek Patkar, Enrico Papini

**Affiliations:** ^1^ Endocrinology, Atrius Health, Boston, MA, United States; ^2^ Beth Israel Deaconess Medical Center, Harvard Medical School, Boston, MA, United States; ^3^ Department of Medicine, Harvard Medical School, Boston, MA, United States; ^4^ Endocrinology Unit, Azienda USL-IRCCS di Reggio Emilia, Reggio Emilia, Italy; ^5^ Deontics Ltd, London, United Kingdom; ^6^ Endocrinology and Metabolism Department, Regina Apostolorum Hospital, Albano, Rome, Italy

**Keywords:** thyroid nodule, clinical practice guideline, clinical calculators clinical decision support, computer interpretable guideline, evidence based care, guideline compliance, CDSS

Thyroid nodules are common, predominantly benign, asymptomatic on presentation, and most often remain so (Uppal et al.) (1). Moreover, those that are malignant are principally small low-risk neoplasms with an indolent course and minimal impact on survival (2). Hence, most patients do not benefit from extensive evaluation, treatment, and monitoring (2–5). On the contrary, costly diagnostic techniques and treatment may have a detrimental impact on a patient’s physical, emotional, and financial status (Uppal et al.). In the United States well over 500 000 fine-needle aspirations (FNAs) of thyroid nodules are performed yearly with as many as 40% likely unnecessary (6). In European countries, such as Germany and France, as well as well as in the United States, overtreatment is reflected by most thyroidectomies performed for nodular thyroid disease prove to be for benign disease while the minority that are malignant are principally comprised of low-risk thyroid cancers (7). Recommendations from professional societies such as the American Association of Clinical Endocrinologists (AACE), Associazione Medici Endocrinologi (AME) (2), American Thyroid Association (ATA) (3), European Thyroid Association (ETA) (4), and American College of Radiology (5) for reducing the collective burden of evaluating and treating thyroid nodules and low risk thyroid cancers have had limited impact on achieving this goal (6).

Over the past 3 decades narrative or written clinical practice guidelines (CPGs) have emerged as an increasingly important tool to aid clinicians in managing a host of medical conditions. Guidelines are regularly cited in publications and medical education forums and used as a basis for medical decision making in both clinical and administrative settings. Yet, despite their widespread clinical use, there is substantial room for improvement in the following ways:

- Establishing the cost effectiveness and validity of recommendations, which are often based on expert opinion, retrospective studies, and study populations that are not generalizable.
- Evaluating their impact on patient quality of life
- Routinely disseminating, distributing, and implementing guidelines
- Gauging their implementation by tracking their use and applicability
- Creating mechanisms for vetting guideline recommendations in various clinical situations and across different populations and cultures
- Addressing their often-formidable length and the wealth of information they contain, which makes them hard for physicians to navigate as well as absorb and retain (internalize)
- Providing timely updates of narrative multi-authored, highly validated documents

Advanced Clinical Decisions Support Systems (CDSS) addresses all the above-mentioned points by transforming CPGs into computer interpretable guidelines (CIGs). CIGs are derived from CPGs. They employ execution engines (programs) to analyze patient-specific data to electronically generate, document, and track recommendations (Garber and Patkar).

The articles in this Research Topic of Frontiers in Endocrinology are written by a diverse group of authors representing various specialties and regions. They cover the gamut of tools available for evaluating thyroid nodules. They also underscore the challenges in developing a streamlined, cost-effective approach that minimizes unnecessary evaluation and intervention that not only does not benefit patients but may harm them while maximizing the chances for identifying clinically significant disease that if left untreated would lead to significant morbidity.

The diagnostic tools available to clinicians caring for patients with thyroid nodules can be summarized in **Table 1**. Over time, history and physical examination have taken on a marginal role in evaluating thyroid nodules. This is due to several reasons. Many nodules are discovered incidentally on imaging that is not being performed to evaluate the thyroid gland. Since physical examination is not as reliable as ultrasound in establishing the presence, size, or characteristics of benign or malignant disease it is less frequently or carefully performed. Thus, it is not regularly used to follow patients with benign, inconsequential nodularity. This is not without a downside. Greater reliance on ultrasound as a monitoring tool and to facilitate fine needle aspiration of a candidate nodule leads to surveying the remainder of the thyroid gland for nodularity. While improving the yield and accuracy of fine needle aspiration, ultrasound often leads to the detection and evaluation of clinically inapparent nodules that are either diagnostically indeterminate or an inconsequential malignancy, resulting in surgery without clear benefit.

**Table 1:** Diagnostic Tools.

*History (see TNAPP)	
Radiation
Family History
Symptoms
Thyroid Disease
Disorders of function
Structural abnormalities
*Physical Exam
Initial
Serial
Laboratory determinations
*TSH levels
Calcitonin levels
Imaging:
*Ultrasound
*Standard B mode imaging
Elastography
Contrast Enhanced Ultrasound
**Artificial Intelligence
**Computer Aided Diagnosis
**Radiomics
Nuclear medicine
Radioactive Iodine
PET (FDG)
*Clinical Practice Guidelines (CPGs)
*Risk Score Stratification Tools (Clinical Calculators)
Computer Interactive Guidelines
*Fine Needle Aspiration
*Molecular Marker/Diagnostics (mostly USA)
DNA (Mutations)
Messenger RNA
micro– RNA
Immunocytochemistry
*Principal
**Emerging

The current emphasis on evaluating thyroid nodules in addition to standard B-mode ultrasonography, are risk stratification tools such as ACR TIRADS, fine needle aspiration with Bethesda classification and molecular genetic markers (Patel et al.), where available. Additional tools that have not yet been standardized, widely adapted, or extensively studied, include ultrasound elastography (Li et al.), contrast enhanced ultrasound (Zhou et al.), emerging AI tools (Xu D. et al.) (8), and immunocytochemistry (Crescenzi and Baloch; Taccogna et al.). On a positive note, therapeutic advances promoted by professional societies (9–12) employing minimally invasive ablation procedures have been made. These techniques are being used more frequently. Compared with surgery, they are less expensive, cause less morbidity, and have fewer adverse effects on patient quality of life.

Challenges, however, remain. Tools for assessing risk vary, employing different characteristics underpinning a strong argument for universally accepted risk stratification tools (Majety et al.) (13, 14). Oftentimes, newer technology is complementary rather than substitutive, increasing cost without offering consistently substantial benefit (Uppal et al.). Yet reliable, relatively expensive, new AI tools may ultimately play a key role in resource poor regions that not only lack access to diagnostic tools, but do not have the professional expertise to interpret ultrasound (8).

A promising new development strongly supported by the editors of this Research Topic is the adaptation of CIGs to complement and facilitate the use of clinical practice guidelines and risk stratification systems. Using advanced CDSS to co–develop CIGs that complement conventional society guidelines, may not only increase the use of CPGs, but could serve as testing tools for assessing the efficacy and generalizability of a sequence of diagnostic tests (Triggiani et al.) (15). This could be accomplished by employing a range of assumptions and models for the respective sensitivity, specificity, accuracy, and costs of each tool being employed.

Our challenge is improving our approach to evaluating and managing thyroid nodules by increasingly employing minimally invasive techniques, and developing more specific, less costly molecular tests that not only diagnose malignancy but also provide prognostic information. Doing so will substantially reduce the number of preventable adverse effects of invasive diagnostic and non–surgical therapeutic procedures, surgical morbidity, and financial toxicity at the expense of only detecting the relatively small percentage that prove to be thyroid cancers that require treatment.

## Author contributions

JG: Writing – original draft, Writing – review & editing. AF: Writing – review & editing, Writing – original draft. VP: Writing – review & editing. EP: Writing – review & editing.
